# Associations of sense of coherence and self-efficacy with health status and disease severity in COPD

**DOI:** 10.1038/s41533-020-0183-1

**Published:** 2020-06-18

**Authors:** Ioanna Tsiligianni, Dimitra Sifaki-Pistolla, Irini Gergianaki, Maria Kampouraki, Polyvios Papadokostakis, Ioannis Poulonirakis, Ioannis Gialamas, Vasiliki Bempi, Despo Ierodiakonou

**Affiliations:** 1 Health Planning Unit, Department of Social Medicine, Faculty of Medicine, University of Crete, Herkalion, Crete Greece; 2 Clinic of Social and Family Medicine, Faculty of Medicine, University of Crete, Crete, Greece; 3 Moires Health Care Center, Herkalion, Crete Greece; 4 Primary Care Practice, Garipa, Herkalion, Crete Greece; 5 Primary Care Practice, Health Center of Agia Varvara, Heraklion, Crete Greece; 6 Primary Care Practice, Health Center of Sitia, Sitia General Hospital, Lasithi, Crete Greece; 7 Heraklion University Hospital, Heraklion, Crete Greece

## Abstract

Sense of coherence and self-efficacy has been found to affect health-related quality of life in chronic diseases. However, research on respiratory diseases is limited. Here we report findings on quality of life (QoL) of COPD patients and the associations with coherence and self-efficacy. This study consists of the Greek national branch of the UNLOCK study, with a sample of 257 COPD patients. Coherence and self-efficacy are positively inter-correlated (Pearson rho = 0.590, *p* < 0.001). They are negatively correlated with the quality of life (CAT) [Pearson rho: coherence = −0.29, *p* < 0.001; self-efficacy = −0.29, *p* < 0.001) and mMRC (coherence = −0.37, *p* < 0.001; self-efficacy rho = −0.32, *p* < 0.001)]. Coherence is inversely associated with (Global Initiative for Chronic Obstructive Lung Disease) GOLD 2018—CAT and GOLD 2018—mMRC classification and “having at least one exacerbation in the past year”. Findings are stressing the need for their incorporation in primary health care and COPD guidance as it maybe that enhancing coherence and self-efficacy will improve QoL.

## Introduction

Chronic obstructive pulmonary disease (COPD) is one of the leading public health problems worldwide, with severe impact on the health care system and patient’s life^1–3^. COPD patients have high morbidity, disability and mortality rates, with often impaired quality of life, exacerbations and hospital admissions^1,4,5^ COPD is largely irreversible, and it has adverse effects in health-related quality of life (QoL) of those patients^1^. Enhancing self-management could optimize QoL, reduce hospital admissions and contribute to overall disease management^1^.

QoL has been proved extremely important for the management of patients with COPD, that is why Global Initiative for Chronic Obstructive Lung Disease (GOLD) suggested COPD treatment and management to be based on QoL and exacerbations^1^. Although there is a plethora of studies on COPD, there is still paucity of research on what influences QoL of patients with COPD, with a meta-analysis of Tsiligianni et al. suggesting that depression is a leading factor^6^. Further reports emphasize that worse ability to cope with the disease may deteriorate QoL and limit self-management/self-efficacy^6–9^. Adverse influence on QoL could be mitigated by protective factors of stress-coping mechanisms and a better way of managing life events; nowadays research has shifted focus from risk factors to protective mechanisms, positive personal/interpersonal strengths and psychosomatic approaches on managing illness and enhancing QoL^10^. Characteristically, the assessment of sense of coherence (thus how people view their life and how they use their resistance resources to maintain and develop their health) as well as self-efficacy (how people influence their own motivation/action) and their impact on QoL is considered essential for identifying those patients in need of more support for maintaining and building personal and interpersonal strengths^10^. Yet, relevant research on COPD patients is scarce^11^.

In particular, sense of coherence was conceptualized by Antonovsky^12^ and determined as a “global orientation that expresses the extent to which one has a pervasive, enduring though dynamic feeling of confidence” into measurable indicators, namely comprehensibility, manageability and meaningfulness. Interestingly, the two first indicators are included, but only partially and not in a standardized way, in the recommendations on COPD self-management and patient empowerment^1,13^. The associations between QoL and coherence have been extensively studied in the general population^14^, as well as in patients with cardiovascular diseases, breast cancer and mental health problems^15^, whereas studies in patients with respiratory diseases or COPD are limited. This literature outlines the positive impact of coherence on self-efficacy and the strong interactions with better QOL in COPD patients^16,17^. In addition, COPD patients with psychiatric and/or psychological problems may have better QoL and therefore effective treatment, when self-efficacy and coherence are empowered^16,17^.

Self-efficacy is derived from social learning theory and attempts to explain the common mechanism through which people exercise influence over their own motivation and behaviour and thus offers an appealing link between self-perception and individual actions^18^. It is defined as “the degree of confidence that individuals have in their ability to perform specific activities successfully”^18^. A strong sense of self-efficacy is necessary for a sense of personal wellbeing^19^.

The aforementioned are of special interest, especially for further research on COPD patients residing in socially/economically vulnerable environments that could increase exposure to stressors and complex accessibility to health care services, self-management/self-efficacy, adherence to treatment and social support^20,21^. The current study aimed to assess QoL of COPD patients and explore associations with several psychopathological factors, focussing on the role of coherence. Particularly, two major research questions were assessed: To what extent is coherence associated with health status and disease control in COPD patients? and To what extent self-efficacy of COPD patients is associated with health status and disease control?

## Results

### COPD patient characteristics

Mean (SD) population age was 65 (12.3) years with 204 (79.4%) males. Table 1 presents the patients characteristics in terms of exacerbations, QoL and GOLD classification.

**Table 1:** Population characteristics, health status, exacerbations and classification of 257 COPD patients.

QoL, exacerbations and GOLD classification health status	Total *N* = 257
CAT score; mean (SD)	17.2 (6.7)
CAT score ≥10; *n* (%)	224 (91.1)
mMRC score; mean (SD)	1.9 (1.2)
mMRC score ≥2; *n* (%)	154 (60.6)
Number of exacerbations in the past 12 months; median (min.–max.)	1 (0–4)
≥1 exacerbations in the last 12 months; *n* (%)	179 (86.5)
Number of hospitalizations in the past 12 months; median (min.–max.)	0 (0–3)
Hospitalized in the past 12 months; *n* (%)	12 (5.2)
GOLD 2018—CAT; *n* (%)	
A	11 (5.4)
B	115 (56.4)
C	3 (1.5)
D	75 (36.8)
GOLD 2018—mMRC; *n* (%)	
A	57 (27.5)
B	71 (34.3)
C	20 (9.7)
D	59 (28.5)

*BMI* body mass index, *GOLD 2018* Global Initiative for Obstructive Lung Disease 2018 Guidelines, *CAT* Chronic Obstructive Pulmonary Disease Assessment Test, *mMRC* Modified Medical Research Council Dyspnoea Scale. *Adapted from reference*^39^

### Trends coherence and self-efficacy in COPD

Both coherence and self-efficacy were normally distributed and were positively correlated (Pearson rho = 0.590, *p* < 0.001). Sense of coherence sum ranged from 70 to 203 with mean (SD) 150 (21.9). Self-efficacy sum ranged from 12 to 40 with mean (SD) 31 (4.75). Both coherence and self-efficacy were negatively correlated with CAT (Pearson rho for coherence = −0.29, *p* < 0.001; for self-efficacy = −0.29, *p* < 0.001) and mMRC (Pearson rho for coherence = −0.37, *p* < 0.001; for self-efficacy rho = −0.32, *p* < 0.001). CAT and mMRC were positively correlated (rho = 0.55, *p* < 0.001).

### Coherence and self-efficacy associations with COPD outcomes

Table 2 shows the associations of coherence with health status and disease severity in COPD patients. After adjustments, coherence was significantly associated with CAT (beta (95%) −0.06 (−0.10; −0.02)) and mMRC (beta (95%) −0.01 (−0.02; −0.008)), e.g. increase in coherence improved CAT and mMRC scores; for each unit increase in self-efficacy, CAT decreased on average by 0.6 and mMRC by 0.02. When we tested associations with individual CAT items, higher coherence was significantly associated with improvement in scores for breathlessness, activities at home, sleep quality and energy level. Coherence was also associated with lower risk for exacerbations in the past year (odds ratio (OR) (95%) 0.97 (0.95; 0.99). There was also a negative association of coherence with GOLD 2018—mMRC status (OR (95%) 0.97 (0.95; 0.99) and borderline association with GOLD 2018—CAT (OR (95%) 0.97 (0.93; 1.00)). Sense of coherence was not associated with hospitalization due to respiratory illness (OR (95%) 0.10 (0.96; 1.02)).

**Table 2:** Associations of sense of coherence with quality of life, disease severity and classification in COPD patients.

	Beta^a^ or OR^b^ (95% CI)*	*p* Value*
CAT score^a^	−0.06 (−0.10; −0.02)	0.002
mMRC score^a^	−0.01 (−0.02; −0.008)	<0.001
CAT ≥10 vs <10^b^	0.98 (0.95; 1.00)	0.114
mMRC ≥2 vs <2^b^	0.97 (0.99; 0.96)	0.001
Exacerbations; yes vs no^b^	0.97 (0.95; 0.99)	0.015
Hospitalizations; yes vs no^b^	0.10 (0.96; 1.02)	0.412
GOLD 2018—CAT-based^b^ BD vs AC	0.97 (0.93; 1.00)	0.055
GOLD 2018—mMRC-based^b^ BD vs AC	0.97 (0.95; 0.99)	<0.001

*GOLD 2018* Global Initiative for Obstructive Lung Disease 2018 Guidelines, *CAT* Chronic Obstructive Pulmonary Disease Assessment Test, *mMRC* Modified Medical Research Council Dyspnoea Scale, *OR* odds ratio, *95% CI* 95% confidence interval.

*Multivariate linear^a^ or logistic^b^ regression models adjusted for gender, age, smoking status and number of comorbidities.

After adjustments, self-efficacy was significantly associated with CAT (beta (95%) −0.20 (−0.38; −0.01)) and mMRC (beta (95%) −0.05 (−0.90; −0.02)), e.g. increase in self-efficacy improved CAT and mMRC scores; for each unit increase in self-efficacy, CAT decreased on average by 0.2 and mMRC by 0.05. When we tested associations with individual CAT items, higher self-efficacy was significantly associated with improvement in scores for activities at home, confidence leaving home and sleep quality, with a borderline significant association with energy level (data not shown). Self-efficacy was not associated with having at least 1 exacerbation (OR (95%) 0.91 (0.81; 1.02) or hospitalization in the past year due to respiratory illness (OR (95%) 0.93 (0.81; 1.06)). There was an inverse association of coherence with GOLD 2018—CAT (OR (95%) 0.80 (0.66; 0.97) but not with GOLD 2018—mMRC status (OR (95%) 0.95 (0.88; 1.02)) (Table 3). When we added both Sense of Coherence (SOC) Scale and General Self-Efficacy (GSE) Scale, only SOC Scale remained significant (Table 4).

**Table 3:** Associations of self-efficacy with health status, disease severity and classification in COPD patients.

	Beta^a^ or OR^b^ (95% CI)*	*p* Value*
CAT score^a^	−0.20 (−0.38; −0.01)	0.037
mMRC score^a^	−0.05 (−0.90; −0.02)	0.001
CAT ≥10 vs <10^b^	0.90 (0.78; 1.02)	0.112
mMRC ≥2 vs <2^b^	0.96 (0.90; 1.03)	0.234
Exacerbations; yes vs no^b^	0.91 (0.81; 1.02)	0.099
Hospitalizations; yes vs no^b^	0.93 (0.81; 1.06)	0.260
GOLD 2018—CAT-based^b^ BD vs AC	0.80 (0.66; 0.97)	0.027
GOLD 2018—mMRC-based^b^ BD vs AC	0.95 (0.88; 1.02)	0.173

*GOLD 2018* Global Initiative for Obstructive Lung Disease 2018 Guidelines, *CAT* Chronic Obstructive Pulmonary Disease Assessment Test, *mMRC* Modified Medical Research Council Dyspnoea Scale, *OR* odds ratio, *95% CI* 95% confidence interval.

*Multivariate linear^a^ or logistic^b^ regression models adjusted for gender, age, smoking status and number of comorbidities.

**Table 4:** Independent associations of sense of coherence and self-efficacy with health status, disease severity and classification in COPD patients.

	Coherence		Self-efficacy	
	Beta^a^ or OR^b^ (95% CI)*	*p* Value*	Beta^a^ or OR^b^ (95% CI)*	*p* Value*
CAT score^a^	−0.04 (−0.08; −0.001)	0.044	−0.18 (−0.38; −0.018)	0.075
mMRC score^a^	−0.01 (−0.02; −0.004)	0.003	−0.03 (−0.07; 0.006)	0.106
CAT ≥10 vs <10^b^	0.99 (0.96; 1.02)	0.383	0.93 (0.80; 1.09)	0.378
mMRC ≥2 vs <2^b^	0.97 (0.95; 0.91)	0.001	1.02 (0.95; 1.10)	0.568
Exacerbations; yes vs no^b^	0.97 (0.95; 1.00)	0.063	0.96 (0.85; 1.09)	0.550
Hospitalizations; yes vs no^b^	0.99 (0.96; 1.03)	0.673	0.90 (0.77; 1.04)	0.144
GOLD 2018—CAT-based^b^ BD vs AC	0.99 (0.94; 1.03)	0.485	0.85 (0.67; 1.08)	0.186
GOLD 2018—mMRC-based^b^ BD vs AC	0.97 (0.95; 0.99)	0.001	1.02 (0.93; 1.12)	0.656

*GOLD 2018* Global Initiative for Obstructive Lung Disease 2018 Guidelines, *CAT* Chronic Obstructive Pulmonary Disease Assessment Test, *mMRC* Modified Medical Research Council Dyspnoea Scale, *OR* odds ratio, *95% CI* 95% confidence interval.

*Multivariate linear^a^ or logistic^b^ regression models adjusted for gender, age, smoking status and number of comorbidities.

## Discussion

Our study revealed the levels of sense of coherence and self-efficacy, which were also positively intercorrelated. Summarizing major findings as a response to the objectives set by the authors, both outcomes (i.e., coherence, self-efficacy) were inversely correlated with CAT and mMRC. In addition, multivariate effects of several indicators to coherence and self-efficacy were identified. Sense of coherence was inversely associated with CAT, mMRC, GOLD 2018—CAT and GOLD 2018—mMRC status and “having at least one exacerbation in the past year”, while there was no association with hospitalization due to respiratory illness. Lastly, self-efficacy was also inversely associated with CAT, mMRC and GOLD 2018—CAT.

The study of Keil et al.^10^ showed that coherence was significantly associated with reduced levels of anxiety and depression as well as lower COPD-related disability, suggesting that coherence could represent a helpful resource as a protective factor that could help people cope with COPD. Further, the Umeå 85+ study assessed the relationship between coherence and 21 chronic illnesses within a population of patients aged ≥85 years and demonstrated a relationship between low coherence and COPD among others^22^. Boeckxstaens et al. found that even very elderly adults with high coherence scores were shown to have lower mortality rates and less functional decline^23^. It is worth mentioning that these trends were independent of multimorbidity, depression, cognition, disability and sociodemographic characteristics^23^.

Literature supports that general self-efficacy may have a positive impact on improving one’s QoL and health status, especially in COPD patients^24^. Self-efficacy, a core construct of self-management^25^, referring to the “confidence people have in their ability to perform actions that are required to deal with particular situations”, is of particular value for COPD, which is often considered a self-inflicted disease; therefore, patients tend to blame themselves for their condition. Although self-efficacy does not coincide with actual capability, it seems to have a leading role on health behaviour and control disease, enhancing QoL^24^. Moreover, COPD patients often loose sense of control over disease and everyday activities, leading to lower self-efficacy^24,26^. The present study showed a strong association between self-efficacy and QoL as well as severity of dyspnoea symptoms. There is plenty of evidence in the literature that supports that the interactions between COPD symptoms that may increase stress/anxiety levels of patients with lower self-efficacy and coherence^26^. This is in line with other reported evidence in the literature that stresses importance of self-efficacy for better clinical outcomes, psychological and social function^9^.

Furthermore, the study of Kaplan et al.^27^ supports that self-efficacy is a significant univariate predictor of 5-year survival; however, when controlling for FEV_1_ in multivariate survival analysis, self-efficacy has only a marginal effect. Still, the same study stresses the positive correlation of self-efficacy and health status of COPD patients as far as exacerbations and dyspnoea symptoms are concerned^27^. This is in line with our findings; nonetheless, additional studies are still needed.

Towards this direction, it seems that higher levels of self-efficacy have been associated with lower levels of breathlessness, lower levels of anxiety and lower levels of depression even in studies that found no significant association between self-efficacy and exacerbations^24^. Thus increasing self-efficacy in patients living with COPD seems to be a crucial point for self-management support.

Importance of QoL in COPD patients has received prompt attention by the scientific community, since it has already been included in patient classification^1^. Apart from the obvious priorities of the primary health care team, i.e. early diagnosis of population at risk, special focus should be given on training patients to recognize the early COPD-related symptoms, avoid risk factors, such as smoking, and encourage visits to a primary health care unit for raising awareness towards protective factors and healthy lifestyle^6–8,28^. Towards this direction, primary health care could prioritize implementation of non-pharmacological interventions that could enhance COPD patients’ self-efficacy, enhance coherence and wellbeing and monitor them at a primary health care level^28^. Oginska-Bulik suggested coherence, self-efficacy/self-management and dispositional optimism could be used as personal resources for the prevention of adverse health outcomes or models that focus on individual responsibility for own health^29^. Primary health care seems to be the right environment to facilitate and promote individual responsibility for own health in the long term.

Plenty of studies have highlighted and attempted to explain the association between coherence, self-efficacy and positive health outcomes, especially in patients with chronic diseases^30–33^. These associations could be explained based on the fact that strong sense of coherence may contribute to better pain-specific outcomes, mainly for constructs relevant to coping (i.e. better pain self-efficacy and less pain dramatizing)^30^. At the same time, coherence is also associated with QoL, general health, vitality and social functioning^30^, while both coherence^22,23^ and self-efficacy^25,27^ have an even stronger impact on health outcomes of early-stage COPD patients who may have less severe symptoms and better sense of pain and self-independence.

The current study, as a part of the UNLOCK (Uncovering and Noting Long-term Outcomes in COPD and asthma to enhance Knowledge) study, is considered a real-life study that enrols patients from usual consultations in primary care. Its major strength is that findings are expected to be applicable to a wider population, since recruitment was independent of the disease severity. At the same time, inclusion of psychopathological indicators such as coherence, self-efficacy and clinical outcomes may add value to the literature exploring protective factors of COPD exacerbations and related QoL.

Still, study’s design (cross-sectional) may hide risk of information bias and lack of assessing causal effects. The fact that the vast majority of patients were males could slightly limit external validity when discussing symptoms and associations in females. Nevertheless, this overrepresentation of males is not considered a bias of our sampling method, as the proportion of males approximates the national estimates of males in Greek COPD population (~70%), and the slightly higher male COPD subjects in the present study may be explained in rural areas^34^. Moreover, statistical analysis was adjusted for gender to manage potential effects of this proportion.

Multilevel analysis could be used if a more general concept was considered, where individuals interact with their social contexts, e.g. the individual persons are influenced by the social groups or contexts to which they belong, and the properties of those groups are in turn influenced by the individuals who make up that group. An advantage of this class of multilevel models is the ability to estimate the cross-level interaction that provides a measure of the joint effect of a variable at the individual level in conjunction with a variable at the group level (e.g. general practitioner (GP) practice/area)^35,36^. However, an important problem in multilevel modelling is what constitutes a sufficient sample size for accurate estimation. Simulation studies recommend using a minimum group size of 30–50 with at least 50 groups to produce valid estimates for multilevel regression models, and these estimates are further influenced by the prevalence of the outcome^35,36^. Unfortunately, our study, like previous studies, was not designed for such modelling, therefore its sample size (*n* = 257; 53 facilities with participants ranging between 5 and 20 patients per area) was not enough for using two-level analysis. Larger studies taking into account multilevel modelling are required in the future for answering research question of such nature.

Compared to the general population, coherence and self-efficacy have only sparsely been studied in COPD, especially in socially/economically vulnerable populations. This study conveyed new knowledge on the current trends of coherence, self-efficacy and illness severity in the rural population of Crete, Greece during a rather challenging period (economical recession). The observed associations between coherence and self-efficacy, as well as between them, and clinical indicators of COPD could enrich current evidence in the literature and support further research on similar settings. These concepts (i.e. coherence, self-efficacy) may foster interdisciplinary approaches within and between psychology, medicine and other behavioural and social sciences, while they could enhance the role of multidisciplinary primary health care teams. Focussing on individual-level coherence and self-efficacy, in the face of a heavily burdening and progressive COPD, could enhance treatments aiming at sustaining or prolonging QoL. Towards this direction, coherence and self-efficacy assessment could be actively incorporated into GPs’ daily practice, either during consultation or in the waiting rooms with the use of standardized tools. Future research shall focus on the development and validation of relevant educational materials and tools. Within this context, comprehensive and effective training of physicians and the primary health care team is required to empower their role in disease management and increase their confidence in demonstrating proper inhaler technique that might influence patient coherence and self-efficacy and therefore overall improved care. Special focus could also be given on physician–patient relationship and physician’s clinical and counselling skills to help COPD patients improve their lifestyle and manage core risk factors, such as smoking. Succeeding in quitting smoking and other life changes will directly empower patients and enhance their confidence, sense of coherence and QoL.

GOLD has already recognized the significance of QoL but is still missing these major concepts of coherence and self-efficacy, which play an integral role in QoL and improvement of symptoms. For instance, the observed inverse associations between coherence and CAT, mMRC, GOLD 2018—CAT and GOLD 2018—mMRC may indicate the value of investing in coherence in order to diminish patients’ symptoms, enhance QoL and could probably result in a re-classification of patients from BD to the better AC.

## Methods

### Study design and setting

This real-life study consists of the Greek national branch of the UNLOCK, an international collaboration between primary care researchers to coordinate and share data sets of relevant diagnostic and follow-up variables for COPD and asthma management in primary care^37^. Among others, research questions considered are patient- and physician-related factors affecting outcomes and standards of care and long-term natural course of COPD in primary care.

A convenience sampling method was used to select COPD patients living in rural and semi-urban areas served by primary care facilities across Greece. A sample of 257 COPD patients, previously diagnosed by spirometry performed by chest physicians, was cross-sectionally enrolled between 2015 and 2016 from 53 primary care facilities in Greece. More details on the methodology are available in previous publications^38,39^. The study was approved by the local medical ethics committee of the University Hospital of Crete Greece (protocol number 7985), while all participants agreed to participate and provided written consent forms.

### Processes and tools

GPs performed structured interviews with the aim to collect cross-sectional information, including demographic characteristics, medical history, lifestyle, CAT^40^ and mMRC^41^ scores, annual number of exacerbations and hospitalizations due to respiratory illness and medication used for COPD management. Extended details on exacerbations, definitions, comorbidities and overall processes and standards are provided in previous study publications^38,39^. For measuring sense of coherence, we used the original SOC questionnaire developed by Antonovsky^12,42^, which was translated and validated into Greek^43^. It consists of 29 items (SOC-29), 11 items measuring comprehensibility, 10 items measuring manageability, and 8 items measuring meaningfulness. The response can receive a score between 1 and 7 points on a Likert scale. SOC is measured with a single total score ranging from 29 to 203 points for the SOC-29 questions. Higher scores indicate greater levels of sense of coherence.

For measuring self-efficacy in COPD subjects, we used the GSE Scale^44^ and particularly the validated Greek version^45^. This scale, comprised of a 10-item scale, was created to assess a general sense of perceived self-efficacy. The total score is calculated by finding the sum of all the items. For this scale, the total score ranges between 10 and 40, with a higher score indicating more self-efficacy.

### Statistical analysis

Descriptive statistics for baseline data were presented as percentage or mean and standard deviation (SD). If the distribution of continuous data was not normal, then median (minimum–maximum) was used instead. Pearson rho coefficient was used to assess correlations between coherence, self-efficacy and CAT and mMRC scores. With multivariate linear or logistic regression models, we tested for associations of coherence and self-efficacy with health status (CAT and mMRC scores), exacerbations, and hospitalizations, as well as GOLD 2018 ABCD status (BD vs AC), adjusting for age, gender, smoking status and number of comorbidities. A *p* value of < 0.05 was considered to indicate statistically significant associations (two-sided tests). Data management and statistical analyses were performed in the IBM-SPSS Statistics software (version 23).

## Data Availability

Individual-level data used for this study are held within the servers of the UNLOCK study and the Department of Social Medicine and could not be publically available due to privacy reasons. Data are available from the authors on request.

## References

[1] GOLD. Global strategy for the diagnosis, management and prevention of COPD. Global initiative for chronic obstructive lung disease, GOLD 2018 report (2018). https://goldcopd.org/wp-content/uploads/2017/11/GOLD-2018-v6.0-FINAL-revised-20-Nov_WMS.pdf (2018). Accessed 17 Apr 2019.

[2] Lozano R (2012). Global and regional mortality from 235 causes of death for 20 age groups in 1990 and 2010: a systematic analysis for the Global Burden of Disease Study 2010. Lancet.

[3] Hillas G, Perlikos F, Tsiligianni I, Tzanakis N (2015). Managing comorbidities in COPD. Int. J. Chron. Obstruct. Pulmon. Dis..

[4] Tsiligianni I, Kosmas E, Van der Molen T, Tzanakis N (2013). Managing comorbidity in COPD: a difficult task. Curr. Drug Targets.

[5] Tsiligianni I, Rodríguez MR, Lisspers K, LeeTan T, Infantino A (2017). Call to action: improving primary care for women with COPD. npj Prim. Care Respir. Med..

[6] Tsiligianni I, Kocks J, Tzanakis N, Siafakas N, van der Molen T (2011). Factors that influence disease-specific quality of life or health status in patients with COPD: a review and meta-analysis of Pearson correlations. Prim. Care Respir. J..

[7] de Ridder D, Geenen R, Kuijer R, van Middendorp H (2008). Psychological adjustment to chronic disease. Lancet.

[8] Disler RT, Gallagher RD, Davidson PM (2012). Factors influencing self-management in chronic obstructive pulmonary disease: an integrative review. Int. J. Nurs. Stud..

[9] Decramer M (2011). The European Union conference on chronic respiratory disease: purpose and conclusions. Eur. Respir. J..

[10] Keil DC, Vaske I, Kenn K, Rief W, Stenzel NM (2017). With the strength to carry on: The role of sense of coherence and resilience for anxiety, depression and disability in chronic obstructive pulmonary disease. Chron. Respir. Dis..

[11] Delgado C (2007). Sense of coherence, spirituality, stress and quality of life in chronic illness. J. Nurs. Scholarsh..

[12] Antonovsky, A. *Unraveling the Mystery of Health: How People Manage Stress and Stay Well* (US: Jossey-Bass, 1987).

[13] Bringsvor HB (2018). Effects of a COPD self-management support intervention: a randomized controlled trial. Int. J. Chron. Obstruct. Pulmon. Dis..

[14] Giglio RE, Rodriguez-Blazquez C, de Pedro-Cuesta J, Forjaz MJ (2015). Sense of coherence and health of community-dwelling older adults in Spain. Int. Psychogeriatr..

[15] Rohani C, Abedi HA, Sundberg K, Langius-Eklöf A (2015). Sense of coherence as a mediator of health-related quality of life dimensions in patients with breast cancer: a longitudinal study with prospective design. Health Qual. Life Outcomes.

[16] Warwick M, Gallagher R, Chenoweth L, Stein-Parbury J (2010). Self-management and symptom monitoring among older adults with chronic obstructive pulmonary disease. J. Adv. Nurs..

[17] Potoczek A, Nizankowska-Mogilnicka E, Bochenek G, Szczeklik A (2008). Links between panic disorder, depression, defence mechanisms, coherence and family functioning in patients suffering from severe COPD. Psychiatr. Pol..

[18] Bandura, A. *Social Foundation of Thought and Actions: A Social Cognitive Theory* (Englewood Cliffs, NJ, USA: Prentice Hall, 1986).

[19] Fossion P (2014). Disentangling sense of coherence and resilience in case of multiple traumas. J. Affect. Disord..

[20] Tsiligianni IG (2013). COPD patients trapped in the financial crisis in rural Crete. Lancet Respir. Med..

[21] Tsiligianni IG (2014). Impact of the financial crisis on adherence to treatment of a rural population in Crete, Greece. Qual. Prim. Care.

[22] Lövheim H, Graneheim UH, Jonsén E, Strandberg G, Lundman B (2013). Changes in sense of coherence in old age - a 5-year follow-up of the Umeå 85+ study. Scand. J. Caring Sci..

[23] Boeckxstaens P (2016). A high sense of coherence as protection against adverse health outcomes in patients aged 80 years and older. Ann. Fam. Med..

[24] Andenaes R, Bentsen SB, Hvinden K, Fagermoen MS, Lerdal A (2014). The relationships of self-efficacy, physical activity, and paid work to health-related quality of life among patients with chronic obstructive pulmonary disease (COPD). J. Multidisc. Healthc..

[25] Nici L (2006). American Thoracic Society/European Respiratory Society statement on pulmonary rehabilitation. Am. J. Respir. Crit. Care Med..

[26] Bringsvor HB (2018). Effects of a COPD self-management support intervention: a randomized controlled trial. Int. J. Chron. Obstruct. Pulmon. Dis..

[27] Kaplan RM, Ries AL, Prewitt LM, Eakin E (1994). Self-efficacy expectations predict survival for patients with chronic obstructive pulmonary disease. Health Psychol..

[28] Bellamy D, Smith J (2007). Role of primary care in early diagnosis and effective management of COPD. Int. J. Clin. Pract..

[29] Oginska-Bulik N (2005). The role of personal and social resources in preventing adverse health outcomes in employees of uniformed professions. Int. J. Occup. Med. Environ. Health.

[30] Chumbler NR (2013). Association between sense of coherence and health-related quality of life among primary care patients with chronic musculoskeletal pain. Health Qual. Life Outcomes.

[31] Eriksson M, Lindstrom B (2007). Antonovsky’s sense of coherence scale and its relation with quality of life: a systematic review. J. Epidemiol. Community Health.

[32] Silarova B (2012). Sense of coherence as an independent predictor of health-related quality of life among coronary heart disease patients. Qual. Life Res..

[33] Benyamini Y, Roziner I, Goldbourt U, Drory Y, Gerber Y (2013). Depression and anxiety following myocardial infarction and their inverse associations with future health behaviors and quality of life. Ann. Behav. Med..

[34] Papaioannou AI (2014). Sex discrepancies in COPD patients and burden of the disease in females: a nationwide study in Greece (Greek Obstructive Lung Disease Epidemiology and health ecoNomics: GOLDEN study). Int. J. Chron. Obstruct. Pulmon. Dis..

[35] Moineddin R, Matheson FI, Glazier RH (2007). A simulation study of sample size for multilevel logistic regression models. BMC Med. Res. Methodol..

[36] Maas, C. J., & Hox, J. J. Sufficient sample sizes for multilevel modeling. *Methodology* **1**, 86–92 (2005).

[37] Chavannes N (2010). UNLOCK: uncovering and noting long-term outcomes in COPD to enhance knowledge. Prim. Care Respir. J..

[38] Ierodiakonou D (2019). Determinants of frailty in primary care patients with COPD: the Greek UNLOCK study. BMC Pulmon. Med..

[39] Tsiligianni I, Kampouraki M, Ierodiakonou D, Poulonirakis I, Papadokostakis P (2019). COPD patients’ characteristics, usual care, and adherence to guidelines: the Greek UNLOCK study. Int. J. Chron. Obstruct. Pulmon. Dis..

[40] Jones PW (2009). Development and first validation of the COPD Assessment Test. Eur. Respir. J..

[41] Bestall JC (1999). Usefulness of the Medical Research Council (MRC) dyspnoea scale as a measure of disability in patients with chronic obstructive pulmonary disease. Thorax.

[42] Antonovsky A (1993). The structure and properties of the Sense of Coherence Scale. Soc. Sci. Med..

[43] Paika V, Ntountoulaki E, Papaioannou D, Hyphantis T (2017). The Greek Version of the Sense of Coherence Scale (SOC-29): psychometric properties and associations with mental illness, suicidal risk and quality of life. J. Psychol. Clin. Psychiatry.

[44] Schwarzer, R., & Jerusalem, M. in Measures in health psychology: A user’s portfolio. Causal and control beliefs (eds Weinman, J., Wright, S. & Johnston M.) 35–37 (NFER-NELSON, Windsor, 1995).

[45] Mystakidou K, Parpa E, Tsilika E, Galanos A, Vlahos L (2008). General perceived self-efficacy: validation analysis in Greek cancer patients. Support. Care Cancer.

